# Re: Causal analysis of the impact of serum 25-hydroxyvitamin D levels on laryngeal cancer

**DOI:** 10.1016/j.bjorl.2026.101759

**Published:** 2026-02-06

**Authors:** Hinpetch Daungsupawong, Viroj Wiwanitkit

**Affiliations:** a Private Academic Consultant, Phonhong, Laos; b Saveetha University, Saveetha Institute of Medical and Technical Sciences, Saveetha Dental College and Hospitals, Department of Research Analytics, Chennai, India

Dear Editor,

The publication on “Causal analysis of the impact of serum 25-hydroxyvitamin D levels on laryngeal cancer: A two-sample mendelian randomization study”^1^ is interesting. Rhis study employed Mendelian Randomization (MR) to establish a causal link between vitamin D (25(OH)D) levels and laryngeal cancer incidence. This strategy is an advanced approach that addresses the constraints of observational research. However, essential concerns address statistical difficulties, confounding variables, and interpretation, which should be expanded to provide more detailed results.

While using large-scale GWAS data improves reliability, selecting SNPs as Instrumental Variables (IVs) has constraints, specifically identifying whether these SNPs are actually connected with vitamin D and do not directly impact laryngeal cancer (pleiotropy). Using just four MR approaches may be insufficient. MR-PRESSO or non-linear analysis could increase precision. Furthermore, using only summary statistics may not allow for a complete examination of individual heterogeneity or other factors.

Although smoking was included in the multivariable MR analysis, several variables related with both vitamin D and laryngeal cancer were identified, including body mass index and vitamin D-rich food intake. Alternatively, solar exposure differs by geography, as does socioeconomic level, which may influence both health practices and cancer risk. This constraint may preclude the conclusions from clearly establishing causality.

The GO enrichment analysis results, which show that vitamin D levels are related to the regulation of external metabolites and environmental responses, may indicate that vitamin D's role extends beyond the immune system to protect cells from oxidative stress and chronic inflammation, both of which are key mechanisms in the development of laryngeal cancer. This new interpretation allows for more cellular research and may be related to the concept of precision prevention with vitamin D supplementation in at-risk groups.

To further enhance future study, consider the following questions: 1) Do higher-than-normal vitamin D levels genuinely have a protective effect? Is there a saturation point? 2) Do genetic differences amongst groups (e.g., Europeans, Asians, and Africans) have distinct causal effects? 3) How do dietary and pharmaceutical vitamin D supplementation differ in terms of protective effects from sun exposure? 4) How might vitamin D supplementation be included into public health initiatives for laryngeal cancer screening and prevention?

## ORCID ID

Hinpetch Daungsupawong: 0009-0002-5881-2709

## CRediT authorship contribution statement

Hinpetch Daungsupawong: 50% ideas, writing, analyzing, approval.

Viroj Wiwanitkit: 50% ideas, supervision, approval.

## Declaration of Generative AI and AI-assisted technologies in the writing process

The author use language editing computational tool in preparation of the article.

## Funding

There is no funding.

## Data availability

The data that support the findings of this study are available from the corresponding author, Daungsupawong H, upon reasonable request.

## Declaration of competing interest

The authors declare no conflicts of interest.
